# Clinical pathways for Korean medicine: An implementation approach to impact on the clinical process and association with attitudes

**DOI:** 10.1016/j.heliyon.2024.e32060

**Published:** 2024-05-29

**Authors:** Eunhye Hyun, Hyunmin Kim, Hui-Yong Kwak, Dongsu Kim

**Affiliations:** a Institute of Health Policy and Management, Seoul National University Medical Research Center, 103, Daehakro, Jognogu, Seoul, Republic of Korea; b Policy Development Center, National Institute for Korean Medicine Development, 14, Jeongdong-gil, Jung-gu, Seoul, Republic of Korea; c Haneum Neuropsychiatry Clinic of Korean Medicine, 29, Dongmak-ro, Mapo-gu, Seoul, Republic of Korea; d School of Korean Medicine, Dongshin University, 67, Dongshindae-gil, Naju-si, Jeollanam-do, Republic of Korea

**Keywords:** Korean medicine, Korean medicine doctor panel, Clinical pathway, Implementation research

## Abstract

**Background:**

South Korea's Ministry of Health and Welfare has developed clinical pathways for Korean Medicine (KM-CPs). As part of this initiative, a panel comprising Korean Medicine doctors (KMD) was assembled. This implementation study aimed to preliminarily explore how KM-CP implementation affects the appropriateness and efficiency of the clinical process and its relation to attitude.

**Methods:**

Through random sampling, 311 KMDs were recruited as panelists to participate in two surveys. The surveys included information regarding the KM clinical environment and KM-CP implementation. A panel management program and educational materials were provided to KMDs between the two survey periods. Only 262 KMDs who responded to both surveys were included in the analysis. Three analyses were conducted: 1) descriptive analysis of the study variables, 2) panel analysis using the ordered logit regression model to elucidate the impact of KM-CP on the appropriateness and efficiency of the clinical process, and 3) ordered logit regression analysis of the association between KM-CP implementation and attitude.

**Results:**

More than two-thirds of the KMDs attempted to adopt KM-CP, with mostly positive perception expressed by these doctors. However, expectations and concerns coexist with the standardization of KM-CP. Cases in which KM-CPs were *partially* and *mostly implemented* respectively had negative and positive effects on the appropriateness and efficiency of the clinical process compared to those in which KM-CPs were *not implemented*. Compared to *neutral attitude*, *positive and very positive attitudes* tended to be associated with increased implementation of KM-CP. However, statistical significances were not observed.

**Conclusions:**

The impact of KM-CP on the clinical process and its association with attitude were found to be statistically unclear or inconsistent. Considering the study limitations and implications, we suggest a policy and academic strategies aimed at fostering improvement to enhance its utility.

## Highlights

• Physicians' behavior is influenced by their attitudes.
• South Korea's Ministry of Health and Welfare has developed clinical pathways for Korean Medicine (KM-CP), which is official traditional and complementary and alternative medicine of South Korea.
• *Partially and mostly implemented* of KM-CP showed negative and positive effect on the appropriateness and efficiency of clinical process, respectively, compared to *not implemented.*
• Positive attitude toward KM-CP may be related to active implementation.

## 1 Introduction

A clinical pathway (CP) is a tool designed to improve quality, efficiency, and accessibility in patient-centered care. CP primarily began to be used in the early 1900s, predominantly in the United Kingdom and United States [1]; however, the definitions and terminology of CP are found to vary [2]. According to Pearson et al. [3], CP constitutes a patient care plan, essentially a set of guidelines outlining the sequence of actions that should be followed by healthcare personnel to efficiently achieve patient care goals. Hindle et al. [4] defined CP as a document that describes the usual process for providing multidisciplinary care and a record of the actual care provided during the care episode. Through a literature review and consultations with the European Pathways Association, Kinsman et al. [2] identified five criteria for defining CP: multidisciplinary plan, guidelines or evidence, detailed steps, time frames, and standardized care tailored to specific population. Bleser et al. [5] summarized 84 terms for CP, including critical pathway, care pathway, care map, integrated care pathway, protocol, and guidelines. Despite these varying definitions and terms, the overall objective of CP is to enhance the quality and efficiency of the clinical process.

To achieve the objectives of CP, this tool must be actively implemented by its primary users, namely physicians. Consequently, extensive efforts have been undertaken to encourage physicians to implement CP. However, simply providing knowledge on CP is insufficient to change the routine of physicians [6]. According to Cabana et al. [7], physicians follow a ‘Knowledge-Attitude-Behavior’ structure, which indicates that behavior is influenced by attitude. Based on a survey of physicians in 17 EU countries, Hindle et al. [4] identified negative attitudes (e.g., cultural aversion among doctors) as one of the factors that limit the implementation of CP. Evans-Lacko et al. [6] also indicated that negative attitude is a barrier to CP implementation, citing lack of outcome expectancy and unconvincing rationales. Altogether, these findings imply that physicians' attitude toward CP implementation must be considered.

From 2016 to 2022, South Korea's Ministry of Health and Welfare devoted an R&D budget of $26 million to the development of clinical practice guidelines (KM-CPGs) and clinical pathways (KM-CPs) for Korean Medicine (KM). The KM-CPGs were developed for 30 diseases based on the most recent evidence and a consensus among academic and clinical experts [8]. According to the recommendations of the KM-CPGs, KM-CPs were established as specific and frequently employed guidelines for the clinical process [9]. Unlike the conventional CPs developed by individual institutions to fit their respective condition [10], KM-CPs were developed through a government-led and top-down approach to expand KM's health insurance coverage through KM-CPG and KM-CP [11]. As a result, determining whether KM-CP is adequately utilized across various institutions and identifying strategies to encourage its implementation are important objectives. In 2021, the government launched several projects in anticipation of the official release of KM-CP. The establishment of the ‘Korean Medicine doctor (KMD) panel,’ supported by the National Institution for Korean Medicine Development under the Ministry of Health and Welfare, was a project that aimed to test KM-CPG and KM-CP.

This study, as an observational implementation approach [12], establishes a panel comprising KMDs and conducts a survey on KM-CPG and KM-CP. The findings not only provide a general understanding of the clinical environment of KM but also observational information on the implementation and attitude toward evidence-based clinical tools. The final objective of the study is to determine whether the implementation of KM-CP achieved the common goals of CP (Question 1) and elucidate the association between implementation and attitude (Question 2). Herein, we describe the process of establishing the KMD panel and conducting the survey, and answer the following questions.

Question 1 Does KM-CP implementation contribute to improvements in the appropriateness and efficiency of the clinical process?

Question 2 Does KM-CP implementation increase in tandem with a more positive attitude toward KM-CP?

## 2 Methods

### 2.1 Panelist recruitment

**Fig. 1:**
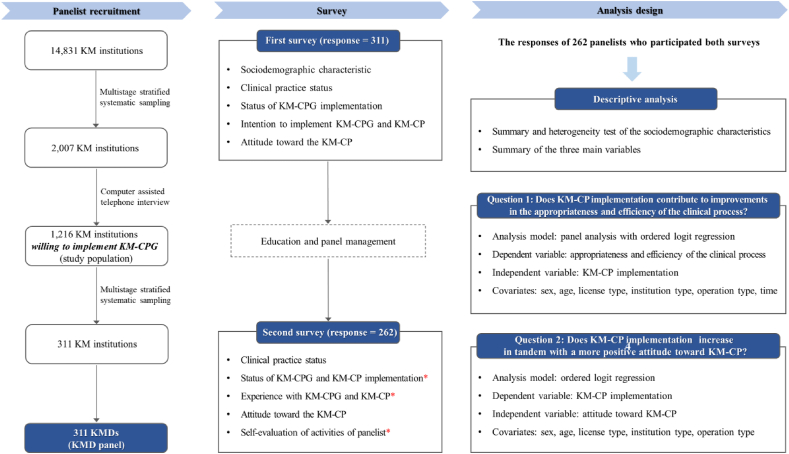
The KMD panelist recruitment process, survey, and analysis design.

The KMD panelist recruitment process, survey, and analysis design are summarized in Fig. 1. KMDs who were willing to implement KM-CPG served as the target population for the panel. KM-CPG (rather than KM-CP) was used as a panel criterion due to the difference in awareness of KM-CPG and KM-CP at the time of panelist recruitment. The preliminary version of the KM-CPG was released in 2018, whereas the KM-CP was introduced in 2022. Therefore, during the panelist recruitment phase in 2020–2021, KM-CPG was relatively more acknowledged. KMDs who were willing to implement KM-CPG were assumed to be the primary users of both KM-CPG and KM-CP. By using a random sampling method, panelists were recruited as follows.

1) The initial panel sampling process was conducted at an institutional level to prevent over-selection of KMDs from a single institution. Initially, all 14,831 KM institutions in South Korea (as of December 2020) were multilevel-stratified based on institution type (hospital or clinic), presence of inpatient beds (yes or no), number of KMDs (one or more), and region (17 provinces). Through proportionate systematic sampling, 2007 of the 14,831 KM institutions were selected as the sample to determine their intention to use the KM-CPG.
2) Computer-assisted telephone interviews were conducted for the 2007 KM institutions to determine their willingness to implement KM-CPG. Of these, 1216 institutions returned positive responses. The representative KMDs from these 1216 institutions served as the panelists for the study. The selected institutions were stratified by institution type (hospital or clinic), presence of inpatient bed (yes or no), number of KMDs (one or more), and region (17 provinces), and systematically sampled.
3) Finally, 311 KM institutions were included in the study and the 311 representative KMDs were recruited to serve as members of the KMD panel.

### 2.2 Survey and data

A total of two surveys were conducted with the panelists. Both these surveys were a combination of online self-administered and interview method using structured questionnaires. The first survey was conducted from October 6, 2021 to November 10, 2021 and comprised 311 respondents. The survey covered the sociodemographic characteristics, clinical practice status, status of KM-CPG implementation, intention to implement KM-CPG and KM-CP, and attitude toward the KM-CP. After the initial survey, in late December 2021, educational materials regarding KM-CPG and KM-CP were provided to the panelists. Panel management was facilitated using letters of appointment, materials, online meetings, and newsletters. The second survey was conducted from February 8 to March 18, 2022 and comprised 262 of the 311 panelists from the first survey (49 panelists were non-respondents). In the second survey, additional questions were included, such as the status of KM-CP implementation, experience with KM-CPG and KM-CP, and self-evaluation of the activities of panelists.

Data collection was carried out by 'Gallup Korea,' the largest and oldest polling institution in Korea. Gallup Korea employs many statistical and survey experts, and receives constant external expert advice on statistics and survey methodology. A professional investigator from Gallup Korea collected the data. The collection process was managed by the supervisor, and the acquired data were verified by a data verification expert. The collected data were edited (verification of errors and omissions on record), coded (encoding and recording of survey contents), and cleaned (error search of input data). The final data processing was performed using the Statistical Package for the Social Sciences (SPSS) program and IBM-compatible Intel Core i5.

### 2.3 Variables

#### 2.3.1 KM-CP implementation

The implementation of KM-CP was determined based on the response to the following question in the second survey: “How much have you utilized the KM-CP since the first survey?” Panelists could select one of four responses regarding KM-CP implementation―*Not implemented, partially implemented, mostly implemented,* and *no patients with the disease*. This process was carried out for a total of 30 diseases(Supplement1), with representative values assigned to the responses for subsequent analysis. Of the four responses, ‘*no patients with the disease*' was treated as a missing value and the remaining responses were used to determine the appropriate representative value. The mean, median, and mode were explored as representative values. Of note, the median can be excessively biased toward *partially implemented* based on the responses to the questions. Although mode is frequently used for categorical variables, multiple values can be selected when the data are evenly distributed. Therefore, mean was selected as the most appropriate representative value.

To calculate the mean of the responses, each response was assigned a score that was re-categorized as an ordinal variable. This process was performed because of the narrow range of the mean value, which would not enable linear regression analysis and dilute the meaning of the original ordinal response. We assigned 1, 2, and 3 points to the three responses: *Not implemented (1), partially implemented (2),* and *mostly implemented (3)* and the mean score for the diseases was calculated per panelist. The mean score was reconverted to the following ordered categories: *Not implemented* (if mean score = 1, indicating KM-CP was not used for any disease despite the availability of patients for its implementation), *partially implemented* (if 1 < mean score≤2), and *mostly implemented* (if 2 < mean score≤3). Finally, KM-CP implementation was used for analysis as an ordinal variable.

#### 2.3.2 Appropriateness and efficiency of the clinical process

The appropriateness and efficiency of the clinical process were assessed using 17 items from both surveys and covered by the question: “How did you feel about the following contents in your clinical process?” The questionnaire was developed according to Jeong et al. [13], who investigated the effectiveness of the clinical application of CP in Korea. In our study, the 'appropriateness and efficiency of clinical process’ was defined as how timely, appropriate, and efficient the test, prescription, procedure or treatment, communication, and work system are performed (detailed questions in Supplement 2). The responses were rated on a 5-point Likert scale: *1 (strongly disagree)–5 (strongly agree)*. The higher the score, the more appropriate and efficient the clinical process.

To summarize the responses measured using the 17 questions, the mean, median, mode, and sum were explored to determine the appropriate representative value. The median may be excessively biased to *neutral*, and the mode may lead to the selection of multiple values when the data are evenly distributed. Finally, in terms of sum, the total value may vary for each respondent due to a lack of response to an item. Therefore, the mean was assumed to be a representative value. However, using the mean as a continuous variable leads to a loss of meaning of the original response measured on the Likert scale. Therefore, the mean was categorized as follows: *very low* (mean = 1), *low* (1 < mean ≤ 2), *neutral* (2 < mean ≤ 3), *high* (3 < mean ≤ 4), and *very high* (4 < mean ≤ 5). The ‘appropriateness and efficiency of the clinical process’ was measured in the two surveys, and the degree of change relates to Question 1.

#### 2.3.3 Attitude toward KM-CP

The attitude toward KM-CP was based on the items selected in the first survey question: “What do you think of the following perceptions of KM-CP?” The draft questionnaire was developed after our discussion regarding "Claimed benefits of clinical pathways" and "Claimed weaknesses of clinical pathways" in Hindle et al. [4]. The questionnaire was revised after consultation with two KMDs and a pilot survey was conducted using a 13-member advisory committee comprising KMDs. This version was finalized after receiving review opinions and performing appropriate revisions.

To ensure content validity, we selected 16 of the 21 items, and reverse-coded some items (No.10–16). The detailed process of item selection and coding is described in(Supplement 3**)**. The responses were rated on a 5-point Likert scale: *1 (strongly disagree)–5 (strongly agree)*. The higher the score, the more positive the attitude of the panelists toward KM-CP.

To summarize the responses measured using the 16 questions (Supplement 4), the mean, median, mode, and sum were explored as representative values. Similar to the reasons provided in '2.3.2 Appropriateness and efficiency of the clinical process,' the mean was used as a representative value. The mean was re-categorized according to the following ordered categories: *very negative* (mean = 1), *negative* (1 < mean ≤ 2), *neutral* (2 < mean ≤ 3), *positive* (3 < mean ≤ 4), and *very positive* (4 < mean ≤ 5).

#### 2.3.4 Covariates

The covariates consisted of five sociodemographic characteristic variables and one time variable. The sociodemographic characteristic variables included sex, age, license type (general practitioner or specialist), institution type (clinic or hospital), and operation type (general or network). These were based on the responses from the first survey and had no missing values. Clinical experience was also associated with the three main variables (**Methods 2.3.1**–**2.3.3**) but was not included as a covariate due to its high correlation with age (Pearson correlation coefficient = 0.88). The time variable indicates the time of the survey (i.e., the first survey or the second survey); it was introduced to adjust for the tendency of each survey point in the panel analysis to be performed on Question 1.

### 2.4 Analysis

The analysis was conducted to (1) provide summary statistics of the study variables, (2) determine whether KM-CP implementation improved the appropriateness and efficiency of the clinical process, and (3) examine the association between KM-CP implementation and attitude. The analysis data consisted of the responses of 262 panelists who participated in both surveys (analysis panel): Of the initial 311 panelists, only 262 were included in the analysis as 49 did not respond to the second survey.

In analyses (2) and (3), the ordered logit regression model was used. However, the estimated coefficient could not be equated to the magnitude of the marginal effect, and the specific magnitude of the marginal effect could be measured through additional prediction probability estimation. This study aimed to offer initial insights into KM-CP, with focus on the overall tendency rather than the effect size.

Few non-responses for items in the questionnaire (<5 %) were excluded from the analysis without imputation [14]. Stata (Version SE/17) was used for the analysis. The significance level was set at 0.05 and 0.01, and the confidence interval was set at 95 %.

#### 2.4.1 Descriptive analysis

Summary statistics (n = 262) were provided for sex, age, clinical experience, license type, institution type, and operation type. Summary statistics of the same variables were also calculated for the entire panelists (n = 311; panelists who were initially selected) and the dropout panelists (n = 49; panelists who participated in the first survey but not the second survey). Heterogeneities among the panel groups were examined using chi-square tests and t-tests. We conducted a descriptive analysis for the three main variables: KM-CP implementation, appropriateness and efficiency of the clinical process, and attitude toward KM-CP.

#### 2.4.2 Question 1: Does KM-CP implementation contribute to improvements in the appropriateness and efficiency of the clinical process?

A panel analysis was conducted using appropriateness and efficiency of the clinical process as the dependent variable, and KM-CP implementation as the independent variable. The covariates included five sociodemographic characteristic variables and one time variable. A fixed-effect model was mainly used to eliminate the influence of unobserved and time-invariant endogenous factors. The analysis was also performed with a random-effect model to consider the uncertainty of the estimation assumption and observe the effects of the time-invariant covariates. An ordered logit regression was employed as the analysis model as the dependent variable was of the ordinal type.

For panel analysis, data from both surveys were required. However, data on KM-CP implementation were only collected in the second survey. Therefore, we assumed that the first survey response of all panelists to KM-CP implementation were '*not implemented’* for the analysis. This assumption may be reasonable as the first survey was conducted before the dissemination of KM-CP to the panelists.

#### 2.4.3 Question 2: Does KM-CP implementation increase in tandem with a more positive attitude toward KM-CP?

An ordered logit regression analysis was conducted using KM-CP implementation as the ordinal dependent variable and the attitude toward KM-CP as the independent variable. The covariates included the five sociodemographic characteristic variables. The time variable was not used as a covariate as a cross-sectional analysis was performed and no adjustment was needed for the tendency over time.

This question was not analyzed using the panel analysis method used for Question 1 **(Methods 2.4.2**) as we could not assume that the first survey data for KM-CP implementation indicate lack of implementation (i.e., survey response: *not implemented*). In fact, all panelists did not implement KM-CP in the first survey due to the study design and not due to their attitude at the time. Therefore, as the next-best option, we performed a cross-sectional regression analysis, considering a time order for the causal relationship between the independent and dependent variables. KM-CP implementation, a dependent variable, was extracted from the second survey; and the attitude toward KM-CP, an independent variable, was extracted from the first survey.

## 3 Results

### 3.1 Summary of the study variables

#### 3.1.1 Sociodemographic characteristic variables and the heterogeneity test

The statistics for the sociodemographic characteristic variables are summarized in Table 1. Heterogeneities with other panel groups were not identified.

**Table 1:** Sociodemographic characteristics of KMD panel.

	Analysis panel (n = 262)		Entire panel (n = 311)		Dropout panel (n = 49)	
	Mean or N	Std or %	Mean or N	Std or %	Mean or N	Std or %
Sex: female	38	14.50	43	13.83	5	10.2
Age (years)	48.23	9.20	48.33	9.08	48.92	1.21
Clinical experience (years)	18.92	9.15	18.91	9.09	18.9	1.27
License type: specialist	24	9.16	30	9.65	6	12.24
Institution type: hospital	11	4.20	11	3.54	0	0
Operation type: network	20	7.63	22	7.07	2	4.08

*p < 0.05, **p < 0.01.

KMD, Korean Medicine doctor; N, The number of frequency; Std, Standard deviation.

#### 3.1.2 Main variables

**Fig. 2:**
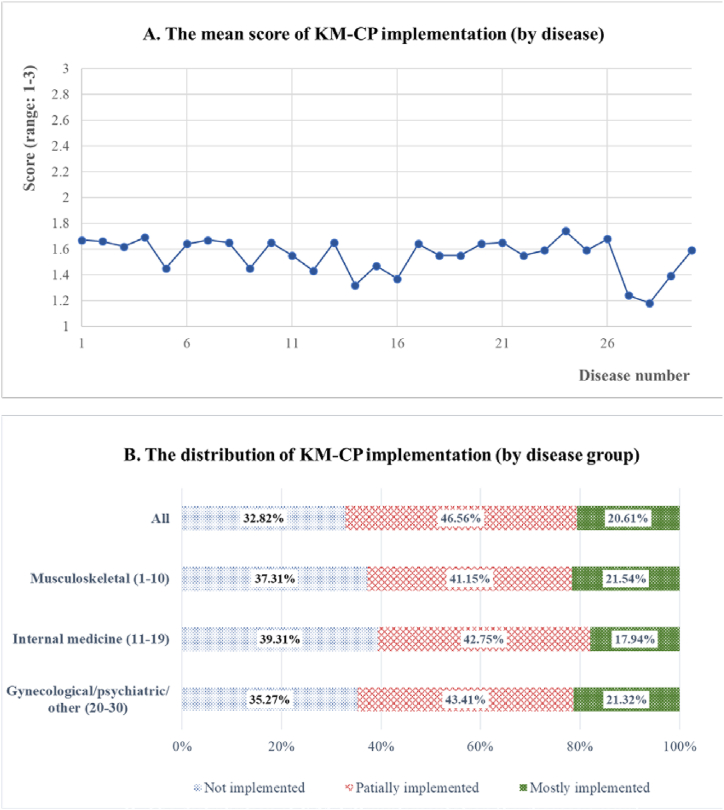
KM-CP implementation.

**KM-CP implementation:** The mean score and distribution of KM-CP implementation in the second survey are presented in Fig. 2(A and B) and Supplement 1. The mean score of all 30 diseases was 1.62 ± 0.6 of 3, with facial nerve palsy (1.74) having the highest score, followed by chronic low back pain (1.69), menstrual pain (1.68), shoulder pain (1.67), and lumbar disc herniation (1.67). The diseases with low scores included autism spectrum disorder (1.18), breast cancer (1.24), cancer-related symptoms (1.32), Parkinson's disease (1.37), and dementia (1.39). For all diseases, KM-CP was *partially implemented*. The proportion of panelists who *mostly implemented* KM-CP was high in the musculoskeletal (21.54 %) and gynecological/psychiatric/other (21.32 %) groups. By defining the sum of the proportions of *partially implemented* and *mostly implemented* as the ‘implementation rate,’ a value of 67.17 % was obtained for all diseases, indicating that more than two-thirds of the analysis panel used KM-CP for at least one disease. Of the disease groups, the highest implementation rate was obtained for the gynecological/psychiatric/other group (64.73 %).

**Fig. 3:**
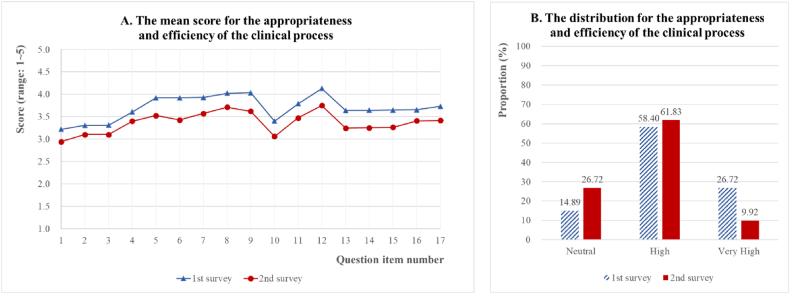
Appropriateness and efficiency of the clinical process.

**Appropriateness and efficiency of the clinical process:** The mean score and distribution for the appropriateness and efficiency of the clinical process in both surveys are provided in Fig. 3(A and B) and Supplement 2. By comparing the results of the two surveys, both the mean scores and each score of the 17 items in the second survey were found to be significantly lower than those obtained in the first survey. All 17 items had a mean score of 3.70 ± 0.56 of 5 in the first survey and 3.37 ± 0.57 of 5 in the second survey. The response tendency of each item was similar in both surveys. A high score was recorded for items related to prescription (No.12) and relationship with patients and caregivers (No.8 and 9), while a low score was recorded for items related to volume and timing of tests (No.1 and 2), timing of prescriptions (No.3), and repetitive non-clinical tasks (No.10). The appropriateness and efficiency of the clinical process was found to be *high* (3 < mean ≤ 4) based on both surveys. Of note, the proportions of *neutral* and *high* increased while that of *very high* decreased in the second survey compared to the first survey.

**Fig. 4:**
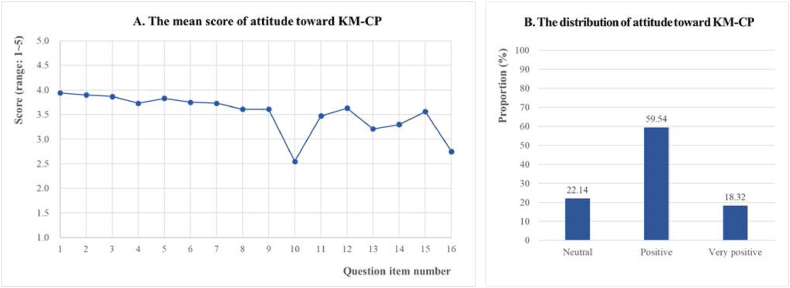
Attitude toward KM-CP

**Attitude toward KM-CP:** The mean score and distribution of attitude toward KM-CP in the first survey are provided in Fig. 4(A and B) and Supplement 4. All 16 items achieved a mean score of 3.53 ± 0.54 of 5. In particular, high scores were assigned to standardization in the clinical process (No.1), efficiency of the clinical process (No.2), quality and outcome of the clinical process (No.3), and teamwork among staff (No.5 and 6), while low scores were assigned for personalized care (No.10) and administrative workload (16). The attitude toward KM-CP was generally *positive* (59.54 %), *neutral* (22.14 %), and *very positive* (18.32 %). No panelist had a *negative* or *very negative* attitude toward KM-CP. By determining the sum of the proportions of *positive* and *very positive* and defining this value as the ‘positive rate,’ 77.86 % of the panelists had a positive attitude toward KM-CP.

### 3.2 Contribution of KM-CP implementation to improvements in the appropriateness and efficiency of the clinical process

The results of panel analysis are presented in Table 2. Based on a fixed-effect model, *partially implemented* KM-CP tended to have a negative effect on the appropriateness and efficiency of the clinical process compared to *not implemented*. *Mostly implemented* KM-CP tended to have a positive effect on the appropriateness and efficiency of the clinical process compared to *not implemented*. However, there were no statistically significant findings. The overall tendency and statistical significance of the random-effect model results were similar to those of the fixed-effect model.

**Table 2:** Panel analysis with ordered logit regression of appropriateness and efficiency of the clinical process.

		All				Disease group											
						Musculoskeletal				Internal medicine				Gynecological/psychiatric/other			
		**FE (n=260)**		**RE (n=262)**		**FE (n=258)**		**RE (n=262)**		**FE (n=260)**		**RE (n=262)**		**FE (n=254)**		**RE (n=258)**	
		**Coef**	**Std**	**Coef**	**Std**	**Coef**	**Std**	**Coef**	**Std**	**Coef**	**Std**	**Coef**	**Std**	**Coef**	**Std**	**Coef**	**Std**
KM-CP implementation (ref. Not implemented)																	
Partially implemented		−0.66	0.50	−0.39	0.35	−0.48	0.50	−0.48	0.34	−0.92	0.50	−0.81^a^	0.34	−0.38	0.51	−0.33	0.35
Mostly implemented		0.31	0.54	0.20	0.43	0.18	0.54	0.14	0.41	0.06	0.56	0.21	0.43	0.15	0.55	0.13	0.42
Covariates																	
	Female (ref. male)	–	–	0.38	0.38	–	–	0.40	0.38	–	–	0.39	0.38	–	–	0.42	0.38
	Age (years)	–	–	0.02	0.01	–	–	0.02	0.01	–	–	0.02	0.01	–	–	0.02	0.01
	Specialist (ref. general practitioner)	–	–	−0.06	0.46	–	–	0.00	0.46	–	–	−0.05	0.46	–	–	−0.10	0.47
	Hospital (ref. clinic)	–	–	1.55^a^	0.66	–	–	1.57^a^	0.67	–	–	1.53^a^	0.66	–	–	1.68^a^	0.68
	Network (ref. general)	–	–	0.51	0.48	–	–	0.38	0.48	–	–	0.52	0.48	–	–	0.54	0.49
	Second survey (ref. first)	−1.15^b^	0.35	−1.13^b^	0.30	−1.20^b^	0.33	−1.11^b^	0.29	−1.01^b^	0.32	−0.97^b^	0.28	−1.29^b^	0.35	−1.20^b^	0.30

FE, Fixed-effect model; RE, Random-effect model; Coef, Coefficient; Std, Standard deviation; ref., reference; KM-CP, Clinical pathway for Korean Medicine.

a p < 0.05.

b p < 0.01.

### 3.3 Association between KM-CP implementation and attitude

The results of the association between implementation and attitude are presented in Table 3. In all analyses, increased positive attitudes toward KM-CP led to higher levels of KM-CP implementation. In particular, KM-CP implementation increased significantly when the attitude was *very positive* compared to *neutral* in the internal medicine and gynecological/psychiatric/other groups.

**Table 3:** Ordered logit regression of KM-CP implementation.

	All (n = 262)		Disease group					
			Musculoskeletal (n = 260)		Internal medicine (n = 262)		Gynecological/psychiatric/other (n = 258)	
	Coef	Std	Coef	Std	Coef	Std	Coef	Std
Attitude toward KM-CP (ref. neutral)								
Positive	0.22	0.29	0.13	0.29	0.32	0.29	0.27	0.29
Very positive	0.70	0.37	0.46	0.37	1.05^b^	0.38	0.79^a^	0.37
Covariates								
Female (ref. male)	−0.37	0.35	−0.42	0.34	−0.41	0.35	−0.43	0.35
Age (years)	−0.01	0.01	−0.01	0.01	−0.01	0.01	−0.01	0.01
Specialist (ref. general practitioner)	−0.14	0.42	−0.12	0.41	0.04	0.43	−0.22	0.44
Hospital (ref. clinic)	−0.15	0.60	0.11	0.64	−0.26	0.62	−0.38	0.61
Network (ref. general)	0.30	0.46	0.40	0.49	0.12	0.45	0.33	0.46

Coef, Coefficient; Std, Standard deviation; KM-CP, Clinical pathway for Korean Medicine.

a p < 0.05.

b p < 0.01.

## 4 Discussion

The rate of KM-CP implementation (67.17 %) by the panelists was similar to the proportion of institutions who expressed their willingness to implement KM-CPG (61 % = 1216/2007 × 100). Therefore, panelists who were willing to use the KM-CPG were assumed to be potential users of the KM-CP. The high score for *mostly implemented* in the musculoskeletal group may be related to the findings of a previous study, in which the development and application of CP were revealed to be easier in diseases with a typical course [15]. KM-CP was also actively implemented in the gynecological/psychiatric/other group. As more panelists had *no patients with the disease* in the gynecological/psychiatric/other group compared to the other two disease groups, many attempts may have been made to use KM-CP for relatively less experienced diseases. Therefore, KM-CP is associated with the common clinical advantage of CP: improvement of the assessment and treatment system [[2], [3], [4], [5]].

The low appropriateness and efficiency for the volume and timing of tests, and the timing of prescriptions can be interpreted in association with the clinical environment of KM institutions. First, if a patient in the KM institution requires a pathology examination or diagnostic imaging test, the patient is often referred to conventional medical institutions. During this process, patients either leave the KM institution or complain of inconvenience. As a result, referrals do not occur at an appropriate volume and time. Second, because only 56 types of herbal medicines are covered by National Health Insurance, most KM prescription fees are not reimbursed. The prescription of non-reimbursed herbal medicines is influenced by non-health factors, such as patients’ financial status, which often makes it difficult to provide herbal medicines in a timely manner [16].

Among attitudes toward KM-CP, ‘standardization of treatment (No.1)’ and ‘personalized treatment (No.10)’ received the highest and lowest ratings, respectively. This difference in interpretation may be due to varying perspectives on the same characteristics of KM-CP. Diagnosis and treatment in KM are centered around a customized approach that is based on the patient's predisposition rather than the disease code. Individuals who view this characteristic as unsystematic anticipate that KM-CP will lead to standardization and reproducibility of KM. Conversely, individuals who embrace this characteristic as a distinctive advantage are concerned that the KM-CP would undermine the advantage of KM.

The tendency of *partially implemented* KM-CP to have a negative effect on the appropriateness and efficiency of the clinical process can be associated with the additional workload required to introduce KM-CP. As more than three-quarters of the panelist institution had fewer than three staff members, and 85 % of the institutions had only one KMD, many institutions may experience difficulty in introducing a new tool, such as KM-CP, from a human resource perspective. In addition, unlike the conventional CP developed by individual institutions to suit their conditions, KM-CP was developed and distributed in a top-down manner. Thus, KM-CP may not reflect the conditions of individual institutions. Accordingly, most panelists may experience remarkable discomfort regarding implementation, leading to a low appropriateness and efficiency of *partially implemented* KM-CP. In contrast, *mostly implemented* KM-CP had a positive effect on the appropriateness and efficiency of the clinical process. Thus, the positive potential of KM-CP for the clinical process is recognized and KM-CP may be effective when actively implemented. Therefore, the KM-CP should be continuously improved to enable more flexible application to various conditions and demands, and support plans should be prepared for its dissemination to institutions.

The association between the attitude toward KM-CP and its implementation was not statistically significant in the musculoskeletal group. In this group, the proportion of panelists that implemented KM-CP, even with a *neutral attitude*, was already high; thus, the margin of increase in implementation with more positive attitude was relatively small compared to those of the other two disease groups. This result is consistent with that of previous studies, which revealed that CP may be easier to implement in conditions with relatively routine prognosis and course, such as musculoskeletal diseases [15]. In contrast, in the internal medicine and gynecological/psychiatric/other groups, attitude had a significant effect on the implementation of KM-CP. Therefore, for diseases in which CP application is relatively difficult, a positive attitude can increase its implementation. In summary, selecting the optimal diseases for the development of KM-CP is not only important, but also a positive attitude toward KM-CP.

This study had some limitations, such as the representativeness of the panel and the generalizability of the results. In this study, the panelists were KMDs that represented KM institutions and expressed willingness to use KM-CPG. Consequently, KMDs who are salaried, do not work at a medical institution, or were not willing to implement KM-CPG, were not represented in this study. Therefore, our results cannot be extrapolated to all KMDs in Korea or professionals in other countries. Second, uncertainty exists regarding measurement. Most of the data relied on the panel's subjective responses, with relatively few objective measurement variables. Nonetheless, not only online self-administered surveys but also interview-type surveys performed by trained professional investigators were conducted to minimize respondent-induced errors. In addition, supervisors were also part of the investigation process to minimize investigator-induced errors. Hence, all procedures for data collection were managed and supervised according to a planned system. Third, the study period was short. Due to time constraints and the limited budget, data on the implementation of KM-CP could only be collected for approximately 2 months. Although short-term pilot results were obtained, follow-up studies reflecting at least 6 months of implementation are needed.

Despite these limitations, this study had the following strengths. First, this study highlights the first attempt to generate empirical data on the implementation of KM-CP by KMDs. Second, long-term improvement and monitoring of related policies can be performed using the panel established in this study. The panel construction method can also be used to lay the foundation for consumer-centered operation of other policies. Third, a strategy can be devised to improve KM-CP. For example, as the impact of KM-CP on the clinical process may vary depending on its degree of implementation, to ensure KM-CP functions in a positive manner, efforts must go beyond simple introduction to active implementation. For active implementation, not only the development of evidence-based but also strategies to secure user-centered convenience and expand positive perceptions are necessary.

The current KM-CP is still in the initial development stage and must be continuously supplemented in the future. More long-term and large-scale studies are needed to assess its performance and factors. Our study serves as the starting point and our results can be used as a reference by policy developers and researchers to improve KM-CP as a flexible tool applicable to variable medical situations and evidence-based standardization.

## 5 Conclusion

In this implementation approach, we confirmed that: (1) more than two-thirds of the panelists attempted to implement KM-CP. (2) Many restrictions exist on the volume and timing of provision in the clinical environment of KM. (3) Most perceptions regarding KM-CP were positive; however, expectations and concerns were found to coexist regarding its standardization. (4) Active implementation of KM-CP can have a positive effect on the clinical process. (5) Positive attitude toward KM-CP may be related to active implementation. However, unclear or inconsistent statistical significances were obtained for the two main study questions. We aim to propose a policy and academic strategy for enhancing the current KM-CP. From a policy perspective, the form and content of KM-CP should be diversified to suit the conditions and demands of individual institutions. In addition, educational programs or regular management systems necessary for KMDs can be introduced to ensure the proper understanding and application of KM-CP. Active publicity is thus needed to improve users' understanding and awareness of KM-CP. From an academic perspective, studies are needed to understand the mechanisms and the attitude of KMDs with regard to detailed KM-CP use. In particular, detailed and quantitative indicators, such as whether a checklist proposed by KM-CP is kept at the institution, whether training for in-house staff is conducted, whether clinical decisions are appropriately set, and whether the goal is achieved, should be established. Additionally, we proposed a qualitative research design to understand the mechanisms by which KM-CP is implemented or avoided. In summary, policy support and multifaceted research efforts will be necessary to enhance the KM-CP.

## Ethics statement

This study was reviewed and approved by the Institutional Review Board of Dongshin University, with the approval number: 1040708-202108-SB-046. All participants provided informed consent to participate in the study.

## Funding

This research received no grant from any funding agency.

## Data availability

The data supporting the findings of this study are available from the corresponding author; however, restrictions apply to the data used under license from the National Institution for Korean Medicine Development. Accordingly, these data are not publicly available. Therefore, data are available from the corresponding author upon reasonable request and with permission from the National Institution for Korean Medicine Development.

The red asterisk (∗) means the additional questions included in the second survey. KM, Korean Medicine; KMD, Korean Medicine doctor; KM-CPG, Clinical practice guideline for Korean Medicine; KM-CP, Clinical pathway for Korean Medicine.

The disease groups were as follows: **(Musculoskeletal group, 1**–**10)** 1. shoulder pain; 2. neck pain; 3. traffic injury; 4. chronic lower back pain; 5. postoperative syndrome; 6. knee pain; 7. lumbar disc herniation; 8. sprained ankle; 9. temporomandibular joint disorders; 10. degenerative spinal stenosis; **(Internal medicine group, 11**–**19)** 11. flu; 12. hypertension; 13. functional dyspepsia; 14. cancer-related symptoms; 15. stroke; 16. Parkinson's disease; 17. migraine; 18. fatigue; 19. vertigo; **(Gynecological/psychiatric/other group, 20**–**30)** 20. menopausal disorder; 21. insomnia; 22. anxiety disorder; 23. cold hands and feet; 24. facial nerve palsy; 25. allergic rhinitis; 26. menstrual pain; 27. breast cancer; 28. autism spectrum disorder; 29. dementia; 30. Hwa-Byung. KM-CP, Clinical pathway for Korean Medicine.

The results of the second survey are presented in (B). The responses for *very low* and *low* are omitted from the figure because they are extremely small compared with the other three categories, with one (0.38 %) and three cases (1.15 %), respectively. The 17 question items are as follows: 1. The number of tests is adequate; 2. The timing of the tests is appropriate; 3. The timing of the prescription is appropriate; 4. The timing of the procedure or treatment is appropriate; 5. There is good communication with nurses; 6. Information is effectively provided to the patients/guardians; 7. The patient/guardian's trust in healthcare staff is high; 8. The relationships between patients/guardians and healthcare staff are good; 9. The self-confidence level in dealing with patients/guardians is high; 10. There are few repetitive tasks that interfere with patient care; 11. Planning of procedures and prescriptions is adequate; 12. Medication is provided without misuse; 13. Patient care system is efficient; 14. Medical record-keeping is efficient; 15. Administrative tasks are rarely missed; 16. The patient care plan is developed and apprehended in advance; 17. Few changes are made to the planned procedures or prescriptions.

Questions 10–16 were analyzed after being rephrased to match the reverse-coded meaning of the original question. The 16 questions were as follows: 1. KM-CP will help reduce and standardize differences in practice among KMDs; 2. KM-CP will help improve practice efficiency; 3. KM-CP can help improve the quality of clinical processes and patient health outcomes; 4. KM-CP will help foster research and innovation to create new treatments; 5. KM-CP will help foster understanding and respect for the entire health workforce; 6. KM-CP will promote teamwork within the health workforce; 7. KM-CP will reduce patient and family anxiety regarding the care process; 8. KM-CP will increase the efficiency of general patient care, allowing more time for difficult patients; 9. KM-CP are developed in government-led projects for efficient management of healthcare costs; 10. KM-CP will not affect personalized care (original question: KM-CP increases the risk that patients with the same disease will receive the same treatment); 11. KM-CP will not increase the risk of a KMD being sued unfairly (original question: KM-CP increase the risk of a KMD being sued unfairly); 12. KM-CP is useful not only for nurses and other health workers, but also for KMDs (original question: KM-CP is useful for nurses and other health workers, but not for KMDs); 13. Autonomy and diversity in clinical processes will be maintained even with KM-CP (original question: KM-CP runs the risk of strictly standardizing care and making hospital management resemble a factory production line); 14. KM-CP is easy to use in hospitals and clinics (original question: KM-CP can only be used in hospitals and is difficult to use in clinics); 15. KM-CP will be used consistently in the future (original question: KM-CP is a current trend that will soon disappear); 16. The administrative workload of KMDs will be similar even with KM-CP (original question: KM-CP will increase the amount of administrative documentation that KMDs need to perform). KM-CP, Clinical pathway for Korean Medicine; KMD, Korean Medicine doctor.

## CRediT authorship contribution statement

**Eunhye Hyun:** Writing – review & editing, Writing – original draft, Methodology, Formal analysis, Data curation, Conceptualization. **Hyunmin Kim:** Writing – original draft, Conceptualization. **Hui-Yong Kwak:** Writing – review & editing, Writing – original draft. **Dongsu Kim:** Writing – review & editing, Writing – original draft, Supervision, Methodology, Investigation, Conceptualization.

## Declaration of competing interest

The authors declare that they have no known competing financial interests or personal relationships that could have appeared to influence the work reported in this paper.
