# Supplementary material for: Clinical pathways for Korean medicine: An implementation approach to impact on the clinical process and association with attitudes
Source: Heliyon. 2024 May 29;10(11):e32060. doi: 10.1016/j.heliyon.2024.e32060 (PMC11176824; doi:10.1016/j.heliyon.2024.e32060)
Supplement: Multimedia component 1 [file mmc1.pptx]

## Slide 1
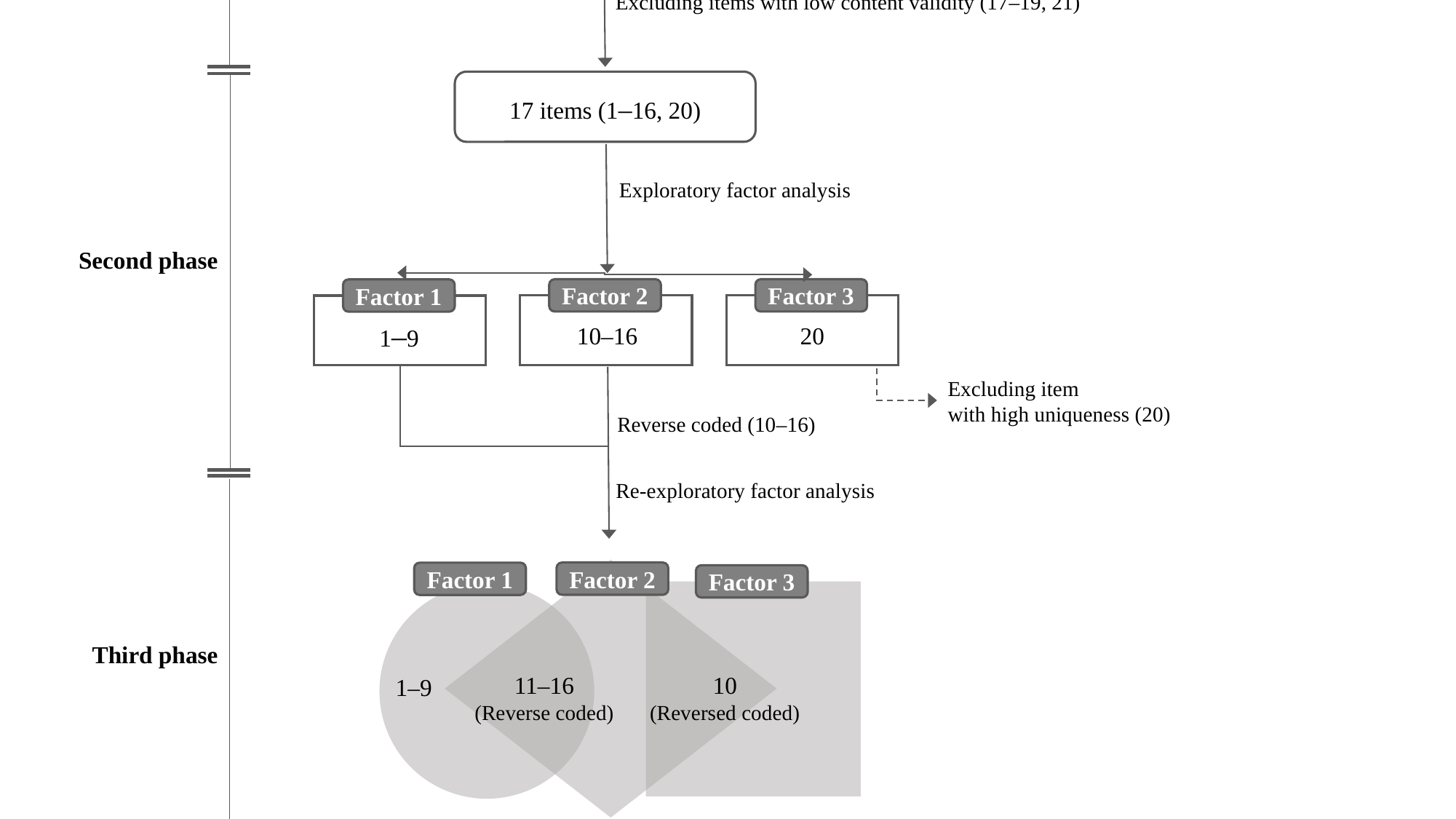

21 items (1–21)
First phase
Excluding items with low content validity (17–19, 21)
17 items (1–16, 20)
Exploratory factor analysis
Second phase
Factor 2
Factor 3
Factor 1
1–9
20
10–16
Excluding item
with high uniqueness (20)
Reverse coded (10–16)
Re-exploratory factor analysis
Factor 2
Factor 1
Factor 3
Third phase
1–9
11–16
(Reverse coded)
10
(Reversed coded)
The 16 items are synthesized as a single-dimensional attitude
