# Supplementary material for: Clinical pathways for Korean medicine: An implementation approach to impact on the clinical process and association with attitudes
Source: Heliyon. 2024 May 29;10(11):e32060. doi: 10.1016/j.heliyon.2024.e32060 (PMC11176824; doi:10.1016/j.heliyon.2024.e32060)
Supplement: Multimedia component 2 [file mmc2.docx]

**Supplement 1. KM-CP implementation**

STable 1. The response score and distribution of KM-CP implementation

|  | | **Response**  **(100%)** |  | **Score (range: 1–5)** | |  | **Distribution (%)** | | |  | **Implementation rate (%)^a^** |
| --- | --- | --- | --- | --- | --- | --- | --- | --- | --- | --- | --- |
|  | |  |  | **Mean** | **Std** |  | **Not implemented** | **Partially implemented** | **Mostly implemented** |  |  |
| All | | 262 |  | 1.62 | 0.60 |  | 32.82 | 46.56 | 20.61 |  | 67.18 |
| By disease group | |  |  |  |  |  |  |  |  |  |  |
|  | Musculoskeletal | 260 |  | 1.66 | 0.65 |  | 37.31 | 41.15 | 21.54 |  | 62.69 |
|  | Internal medicine | 262 |  | 1.58 | 0.61 |  | 39.31 | 42.75 | 17.94 |  | 60.69 |
|  | Gynecological/psychiatric/other | 258 |  | 1.61 | 0.63 |  | 35.27 | 43.41 | 21.32 |  | 64.73 |
| By disease | |  |  |  |  |  |  |  |  |  |  |
|  | 1. Shoulder pain | 257 |  | 1.67 | 0.67 |  | 44.36 | 43.97 | 11.67 |  | 55.64 |
|  | 1. Neck pain | 255 |  | 1.66 | 0.70 |  | 47.06 | 40.00 | 12.94 |  | 52.94 |
|  | 1. Traffic injury | 244 |  | 1.62 | 0.70 |  | 50.41 | 36.89 | 12.70 |  | 49.59 |
|  | 1. Chronic lower back pain | 255 |  | 1.69 | 0.72 |  | 46.27 | 38.82 | 14.90 |  | 53.73 |
|  | 1. Postoperative syndrome | 198 |  | 1.45 | 0.65 |  | 63.64 | 27.78 | 8.59 |  | 36.36 |
|  | 1. Knee pain | 244 |  | 1.64 | 0.70 |  | 49.59 | 37.30 | 13.11 |  | 50.41 |
|  | 1. Lumbar disc herniation | 242 |  | 1.67 | 0.71 |  | 46.69 | 39.26 | 14.05 |  | 53.31 |
|  | 1. Sprained ankle | 251 |  | 1.65 | 0.72 |  | 49.40 | 36.25 | 14.34 |  | 50.60 |
|  | 1. Temporomandibular joint disorders | 196 |  | 1.45 | 0.63 |  | 62.24 | 30.61 | 7.14 |  | 37.76 |
|  | 1. Degenerative spinal stenosis | 246 |  | 1.65 | 0.73 |  | 50.41 | 34.15 | 15.45 |  | 49.59 |
|  | 1. Flu | 228 |  | 1.55 | 0.67 |  | 54.82 | 35.09 | 10.09 |  | 45.18 |
|  | 1. Hypertension | 195 |  | 1.43 | 0.65 |  | 65.64 | 25.64 | 8.72 |  | 34.36 |
|  | 1. Functional dyspepsia | 258 |  | 1.65 | 0.70 |  | 48.06 | 38.76 | 13.18 |  | 51.94 |
|  | 1. Cancer-related symptoms | 154 |  | 1.32 | 0.57 |  | 73.38 | 21.43 | 5.19 |  | 26.62 |
|  | 1. Stroke | 196 |  | 1.47 | 0.66 |  | 62.24 | 28.57 | 9.18 |  | 37.76 |
|  | 1. Parkinson’s disease | 171 |  | 1.37 | 0.61 |  | 70.18 | 22.81 | 7.02 |  | 29.82 |
|  | 1. Migraine | 242 |  | 1.64 | 0.74 |  | 52.07 | 32.23 | 15.70 |  | 47.93 |
|  | 1. Fatigue | 245 |  | 1.55 | 0.69 |  | 56.33 | 32.65 | 11.02 |  | 43.67 |
|  | 1. Vertigo | 232 |  | 1.55 | 0.69 |  | 56.03 | 32.76 | 11.21 |  | 43.97 |
|  | 1. Menopausal disorder | 228 |  | 1.64 | 0.73 |  | 50.88 | 33.77 | 15.35 |  | 49.12 |
|  | 1. Insomnia | 240 |  | 1.65 | 0.73 |  | 50.42 | 34.58 | 15.00 |  | 49.58 |
|  | 1. Anxiety disorder | 215 |  | 1.55 | 0.71 |  | 57.21 | 30.23 | 12.56 |  | 42.79 |
|  | 1. Cold hands and feet | 236 |  | 1.59 | 0.69 |  | 52.97 | 35.59 | 11.44 |  | 47.03 |
|  | 1. Facial nerve palsy | 230 |  | 1.74 | 0.75 |  | 43.91 | 37.83 | 18.26 |  | 56.09 |
|  | 1. Allergic rhinitis | 224 |  | 1.59 | 0.70 |  | 53.13 | 34.82 | 12.05 |  | 46.88 |
|  | 1. Menstrual pain | 225 |  | 1.68 | 0.74 |  | 48.44 | 35.56 | 16.00 |  | 51.56 |
|  | 1. Breast cancer | 135 |  | 1.24 | 0.57 |  | 82.22 | 11.11 | 6.67 |  | 17.78 |
|  | 1. Autism spectrum disorder | 123 |  | 1.18 | 0.46 |  | 85.37 | 11.38 | 3.25 |  | 14.63 |
|  | 1. Dementia | 150 |  | 1.39 | 0.64 |  | 70.00 | 21.33 | 8.67 |  | 30.00 |
|  | 1. Hwa-Byung | 221 |  | 1.59 | 0.70 |  | 53.39 | 34.39 | 12.22 |  | 46.61 |

Note:

1. Implementation rate is defined as the sum of proportions of *partially implemented* and *mostly implemented*.

KM-CP, Clinical pathways for Korean Medicine; Std, Standard deviation.

**Supplement 2. Appropriateness and efficiency of the clinical process**

STable 2-1. The response score of appropriateness and efficiency of the clinical process

|  | | **Response** |  | **Score (range: 1–5)** | | | | |
| --- | --- | --- | --- | --- | --- | --- | --- | --- |
|  | |  |  | **1^St^ survey** | |  | **2^nd^ survey** | |
|  | | **N** |  | **Mean** | **Std** |  | **Mean** | **Std** |
| All (17 items) | | 262 |  | 3.70 | 0.56 |  | 3.37 | 0.57 |
| By item | |  |  |  |  |  |  |  |
|  | 1. The number of tests is adequate. | 262 |  | 3.22 | 0.06 |  | 2.94 | 0.05 |
|  | 1. The timing of the tests is appropriate. | 262 |  | 3.31 | 0.06 |  | 3.10 | 0.05 |
|  | 1. The timing of the prescription is appropriate. | 262 |  | 3.31 | 0.06 |  | 3.10 | 0.05 |
|  | 1. The timing of the procedure or treatment is appropriate. | 262 |  | 3.61 | 0.05 |  | 3.40 | 0.05 |
|  | 1. There is good communication with nurses, | 262 |  | 3.92 | 0.05 |  | 3.53 | 0.05 |
|  | 1. Information is effectively provided to the patients/guardians. | 262 |  | 3.92 | 0.05 |  | 3.43 | 0.05 |
|  | 1. The patient/guardian’s trust in healthcare staff is high. | 262 |  | 3.93 | 0.05 |  | 3.57 | 0.05 |
|  | 1. The relationships between patients/guardians and healthcare staff are good. | 262 |  | 4.02 | 0.04 |  | 3.71 | 0.05 |
|  | 1. The self-confidence level in dealing with patients/guardians is high. | 262 |  | 4.03 | 0.05 |  | 3.62 | 0.05 |
|  | 1. There are few repetitive tasks that interfere with patient care. | 262 |  | 3.40 | 0.06 |  | 3.06 | 0.05 |
|  | 1. Planning of procedures and prescriptions is adequate. | 262 |  | 3.79 | 0.05 |  | 3.47 | 0.05 |
|  | 1. Medication is provided without misuse. | 262 |  | 4.13 | 0.05 |  | 3.75 | 0.05 |
|  | 1. Patient care system is efficient. | 262 |  | 3.64 | 0.05 |  | 3.24 | 0.05 |
|  | 1. Medical record-keeping is efficient. | 262 |  | 3.65 | 0.05 |  | 3.25 | 0.05 |
|  | 1. Administrative tasks are rarely missed. | 262 |  | 3.65 | 0.05 |  | 3.26 | 0.05 |
|  | 1. The patient care plan is developed and apprehended in advance. | 262 |  | 3.66 | 0.05 |  | 3.41 | 0.05 |
|  | 1. Few changes are made to the planned procedures or prescriptions. | 262 |  | 3.73 | 0.05 |  | 3.42 | 0.05 |

N, The number of frequency; Std, Standard deviation.

STable 2-2. The distribution of appropriateness and efficiency of clinical process

|  | | | **Response**  **(100%)** |  | **Distribution (%)** | | | | |
| --- | --- | --- | --- | --- | --- | --- | --- | --- | --- |
|  | | |  |  | **Very low** | **Low** | **Neutral** | **High** | **Very High** |
|  | | | N |  | % | % | % | % | % |
| All (17 items) | | 1st | 262 |  | 0.00 | 0.00 | 14.89 | 58.40 | 26.72 |
|  |  | 2nd | 262 |  | 0.38 | 1.15 | 26.72 | 61.83 | 9.92 |
| By item | | |  |  |  |  |  |  |  |
|  | 1. The number of tests is adequate. | 1st | 262 |  | 2.29 | 16.03 | 48.09 | 24.81 | 8.78 |
|  |  | 2nd | 262 |  | 2.67 | 24.81 | 52.29 | 16.03 | 4.20 |
|  | 1. The timing of the tests is appropriate. | 1st | 262 |  | 1.15 | 15.27 | 46.18 | 26.72 | 10.69 |
|  |  | 2nd | 262 |  | 3.05 | 18.32 | 48.85 | 25.19 | 4.58 |
|  | 1. The timing of the prescription is appropriate. | 1st | 262 |  | 1.15 | 19.47 | 39.31 | 27.48 | 12.60 |
|  |  | 2nd | 262 |  | 1.53 | 19.08 | 51.91 | 22.90 | 4.58 |
|  | 1. The timing of the procedure or treatment is appropriate. | 1st | 262 |  | 0.00 | 9.54 | 36.26 | 38.17 | 16.03 |
|  |  | 2nd | 262 |  | 0.76 | 9.16 | 47.71 | 33.97 | 8.40 |
|  | 1. There is good communication with nurses. | 1st | 262 |  | 0.76 | 1.15 | 28.24 | 45.04 | 24.81 |
|  |  | 2nd | 262 |  | 0.38 | 7.25 | 45.04 | 33.97 | 13.36 |
|  | 6. Information is effectively provided to the patients/guardians. | 1st | 262 |  | 0.00 | 1.53 | 29.39 | 44.66 | 24.43 |
|  |  | 2nd | 262 |  | 0.38 | 9.16 | 46.18 | 35.88 | 8.40 |
|  | 7. The patient/guardian’s trust in healthcare staff is high. | 1st | 262 |  | 0.00 | 1.91 | 26.34 | 48.85 | 22.90 |
|  |  | 2nd | 262 |  | 0.38 | 9.54 | 33.97 | 44.66 | 11.45 |
|  | 8. The relationships between patients/guardians and healthcare staff are good. | 1st | 262 |  | 0.00 | 1.15 | 21.76 | 50.76 | 26.34 |
|  |  | 2nd | 262 |  | 0.38 | 3.44 | 34.73 | 47.71 | 13.74 |
|  | 9. The self-confidence level in dealing with patients/guardians is high. | 1st | 262 |  | 0.00 | 1.53 | 22.90 | 46.18 | 29.39 |
|  |  | 2nd | 262 |  | 0.38 | 7.25 | 34.73 | 45.04 | 12.60 |
|  | 10. There are few repetitive tasks that interfere with patient care. | 1st | 262 |  | 3.82 | 14.12 | 33.59 | 35.11 | 13.36 |
|  |  | 2nd | 262 |  | 3.82 | 19.08 | 48.85 | 24.05 | 4.20 |
|  | 11. Planning of procedures and prescriptions is adequate. | 1st | 262 |  | 0.00 | 6.49 | 29.01 | 43.89 | 20.61 |
|  |  | 2nd | 262 |  | 0.76 | 9.16 | 38.93 | 44.27 | 6.87 |
|  | 12. Medication is provided without misuse. | 1st | 262 |  | 0.00 | 1.91 | 17.18 | 46.95 | 33.97 |
|  |  | 2nd | 262 |  | 0.76 | 6.11 | 30.15 | 43.51 | 19.47 |
|  | 13. Patient care system is efficient | 1st | 262 |  | 0.00 | 6.87 | 37.79 | 39.69 | 15.65 |
|  |  | 2nd | 262 |  | 1.91 | 14.12 | 48.09 | 29.39 | 6.49 |
|  | 14. Medical record-keeping is efficient. | 1st | 262 |  | 0.00 | 7.63 | 36.26 | 40.08 | 16.03 |
|  |  | 2nd | 262 |  | 1.15 | 11.83 | 53.82 | 27.10 | 6.11 |
|  | 15. Administrative tasks are rarely missed. | 1st | 262 |  | 1.15 | 6.11 | 35.50 | 41.22 | 16.03 |
|  |  | 2nd | 262 |  | 1.15 | 13.36 | 48.09 | 32.82 | 4.58 |
|  | 16. The patient care plan is developed and apprehended in advance | 1st | 262 |  | 0.38 | 6.11 | 34.73 | 45.04 | 13.74 |
|  |  | 2nd | 262 |  | 0.76 | 10.69 | 41.60 | 40.84 | 6.11 |
|  | 17. Few changes are made to the planned procedures or prescriptions. | 1st | 262 |  | 0.38 | 4.58 | 32.44 | 46.95 | 15.65 |
|  |  | 2nd | 262 |  | 0.76 | 8.40 | 45.04 | 40.08 | 5.73 |

N, The number of frequencies; Std, Standard deviation.

**Supplement 3. Item selection and coding process for attitude toward KM-CP**

We selected and coded attitude questions toward KM-CP (Clinical pathways for Korean Medicine) by three phases. The entire process is summarized in **SFigure 1**.


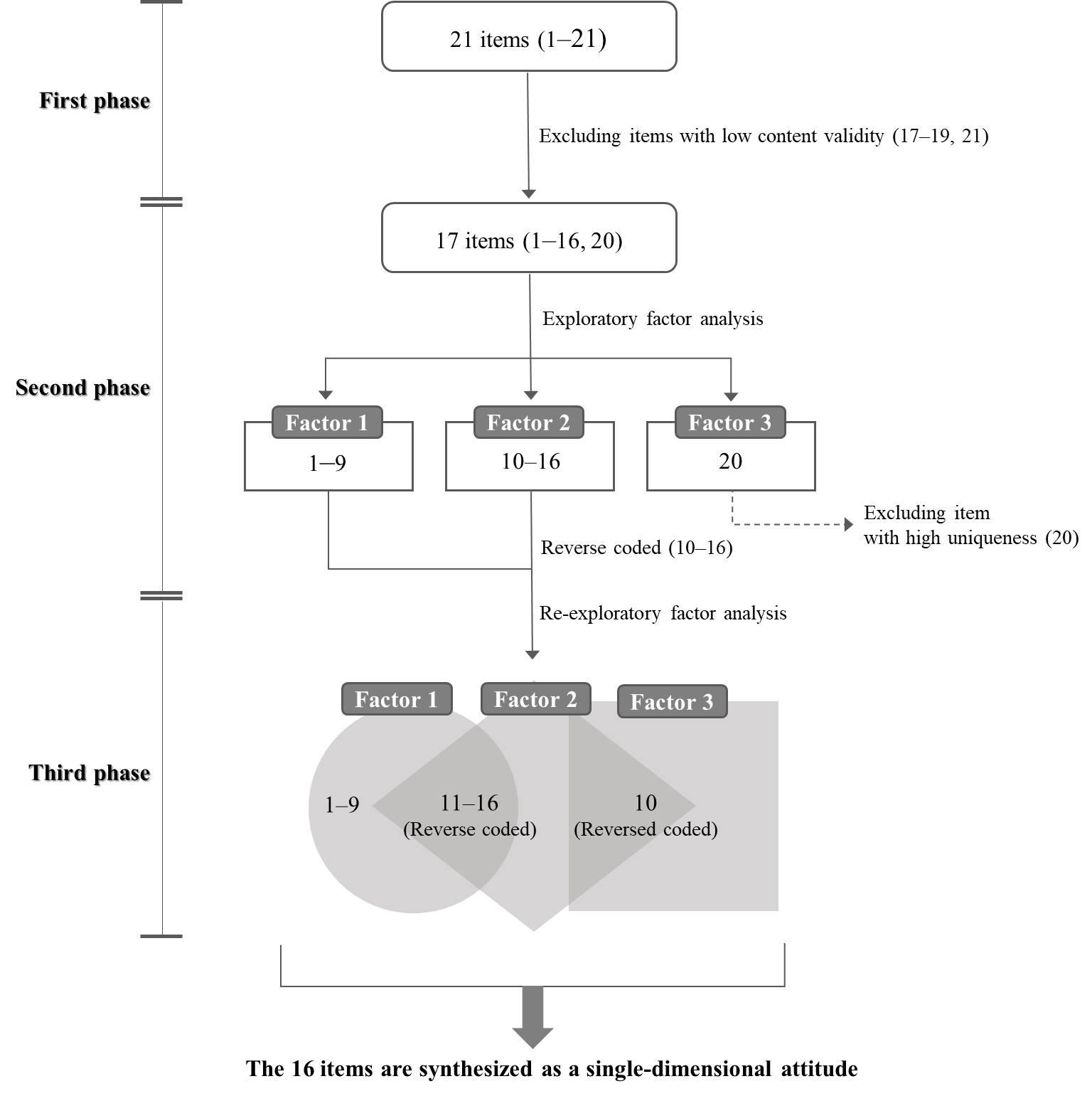


SFigure 1. Item selecting and coding process for attitude toward KM-CP

**First phase:** We excluded the four questions (17–19, 21) that were judged to have low content validity as an attitude toward KM-CP among the 21 original questions. After excluding these four items, the standardized internal consistency (standardized Cronbach’s alpha) increased from 0.86 to 0.89.

*17. At the national level, the needs and importance of healthcare services are increasing, but the funding for healthcare is too small.*

*18. Korean Medicine doctors are not only responsible for health outcomes, but also for cost control.*

*19. Clinical and financial decisions by KMDs have a strong influence on both KMDs and patients.*

*21. A Korean Medicine doctor sometimes needs the opinion of other medical professions in treatment.*

**Second phase:** Exploratory factor analysis based on principal component analysis was performed to determine whether the types of attitudes could be subdivided for the remaining 17 question items (1–16, 20). As a result, as shown in **STable 3-1**, 17 items were classified into three factors: factor 1 (1–9), factor 2 (10–16), and factor 3 (20).

We excluded 20th item from the data source of attitude because the uniqueness of 20th item was 0.52. After that, the standardized Cronbach’s alpha slightly increased from 0.89 to 0.90.

*20. The personal experience of senior clinicians can also be replaced by standardized clinical guidelines.*

On the other hand, the factor loadings of factor 1 and factor 2 generally had a reverse polarity, and factor 1 and 2 could be divided into positive and negative attitudes, respectively. We reverse-coded* the response score of 10-16th items belonging to factor 2 to eliminate the effect of the measurement tool caused by the form of the question (positive or negative questions).

*Reverse coded score = 6 – original score

STable 3-1. Factor loadings and unique variances of factor analysis (1–16, 20)

| **Items** | **Factor 1** | **Factor 2** | **Factor 3** | **Uniqueness** |
| --- | --- | --- | --- | --- |
| 1 | 0.79 | 0.17 | 0.13 | 0.33 |
| 2 | 0.82 | 0.18 | 0.20 | 0.26 |
| 3 | 0.80 | 0.25 | 0.20 | 0.27 |
| 4 | 0.78 | 0.29 | 0.06 | 0.31 |
| 5 | 0.74 | 0.30 | -0.01 | 0.36 |
| 6 | 0.70 | 0.34 | -0.06 | 0.39 |
| 7 | 0.74 | 0.36 | -0.04 | 0.32 |
| 8 | 0.75 | 0.29 | -0.05 | 0.35 |
| 9 | 0.62 | 0.23 | -0.29 | 0.48 |
| 10 | 0.13 | 0.40 | -0.70 | 0.34 |
| 11 | -0.43 | 0.58 | 0.15 | 0.45 |
| 12 | -0.57 | 0.60 | 0.05 | 0.32 |
| 13 | -0.58 | 0.58 | -0.04 | 0.32 |
| 14 | -0.55 | 0.55 | 0.09 | 0.38 |
| 15 | -0.65 | 0.51 | -0.12 | 0.30 |
| 16 | -0.50 | 0.56 | 0.08 | 0.44 |
| 20 | -0.01 | 0.23 | 0.65 | 0.52 |

**Third phase:** Factor analysis was performed again for the remaining 16 items (1–9 and reverse-coded 10-16) in the same way as before, and three factors were as shown in **STable 3-2**. The 1–9 items had strong loading (loadings ≥0.6) in factor 1. Reverse-coded 10 item had strong loading (loadings ≥0.6) in factor 3 and moderate loading (0.4≤ loadings <0.6) in factor 2, respectively. Reverse-coded 11–16 items had moderate or strong loading in both factors 1 and 2. As a result, 1–9 items could be assigned into factor 1, and the reverse-coded 10–16 items could be assigned into factor 2. However, factor 1 also accounted for a significant proportion of reverse-coded 11–16 items.

Therefore, we decided to synthesize 16 question items on attitude towards KM-CP in a single-dimensional attitude without subdividing.

STable 3-2. Factor loadings and unique variances of factor analysis (1–9, reverse coded 10–16)

| **Items** | **Factor 1** | **Factor 2** | **Factor 3** | **Uniqueness** |
| --- | --- | --- | --- | --- |
| 1 | 0.79 | -0.17 | 0.19 | 0.31 |
| 2 | 0.82 | -0.17 | 0.23 | 0.25 |
| 3 | 0.80 | -0.24 | 0.25 | 0.24 |
| 4 | 0.78 | -0.29 | 0.05 | 0.31 |
| 5 | 0.74 | -0.30 | 0.08 | 0.35 |
| 6 | 0.70 | -0.34 | -0.03 | 0.40 |
| 7 | 0.74 | -0.37 | 0.03 | 0.32 |
| 8 | 0.75 | -0.30 | -0.03 | 0.35 |
| 9 | 0.62 | -0.23 | -0.44 | 0.37 |
| 10* | -0.13 | 0.42 | 0.79 | 0.18 |
| 11* | 0.43 | 0.58 | -0.15 | 0.45 |
| 12* | 0.57 | 0.60 | -0.05 | 0.32 |
| 13* | 0.58 | 0.59 | -0.03 | 0.32 |
| 14* | 0.55 | 0.55 | -0.18 | 0.36 |
| 15* | 0.65 | 0.52 | 0.05 | 0.31 |
| 16* | 0.50 | 0.55 | -0.11 | 0.44 |

* Reverse coded

**Supplement 4. Attitude toward KM-CP**

STable 4. The response score and distribution of attitude toward KM-CP

|  | | **Response**  **(100%)** |  | **Score (range: 1–5)** | |  | **Distribution (%)** | | | | |  | **Positive rate (%)^a^** |
| --- | --- | --- | --- | --- | --- | --- | --- | --- | --- | --- | --- | --- | --- |
|  | |  |  | **Mean** | **Std** |  | **Very negative** | **Negative** | **Neutral** | **Positive** | **Very positive** |  |  |
| All (16 items) | | 262 |  | 3.53 | 0.54 |  | 0 | 0 | 22.14 | 59.54 | 18.32 | . | 77.86 |
| By item | |  |  |  |  |  |  |  |  |  |  |  |  |
|  | 1. KM-CP will help reduce and standardize differences in practice among KMDs. | 262 |  | 3.94 | 0.77 |  | 0.38 | 3.05 | 21.37 | 53.05 | 22.14 |  | 75.19 |
|  | 1. KM-CP will help improve practice efficiency. | 262 |  | 3.9 | 0.79 |  | 0.38 | 3.82 | 23.28 | 50.76 | 21.76 |  | 72.52 |
|  | 1. KM-CP can help improve the quality of clinical processes and patient health outcomes. | 262 |  | 3.87 | 0.74 |  | 0.38 | 3.05 | 23.66 | 55.34 | 17.56 |  | 72.90 |
|  | 1. KM-CP will help foster research and innovation to create new treatments. | 262 |  | 3.73 | 0.82 |  | 0.38 | 6.11 | 30.53 | 46.56 | 16.41 |  | 62.98 |
|  | 1. KM-CP will help foster understanding and respect for the entire health workforce. | 262 |  | 3.83 | 0.77 |  | 0.38 | 2.67 | 29.01 | 49.24 | 18.7 |  | 67.94 |
|  | 1. KM-CP will promote teamwork within the health workforce. | 262 |  | 3.75 | 0.79 |  | 0.38 | 2.29 | 37.4 | 41.98 | 17.94 |  | 59.92 |
|  | 1. KM-CP will reduce patient and family anxiety regarding the care process. | 262 |  | 3.73 | 0.82 |  | 0.38 | 6.49 | 29.01 | 47.71 | 16.41 |  | 64.12 |
|  | 1. KM-CP will increase the efficiency of general patient care, allowing more time for difficult patients. | 262 |  | 3.61 | 0.81 |  | 0.38 | 6.87 | 37.4 | 42.37 | 12.98 |  | 55.34 |
|  | 1. KM-CP are developed in government-led projects for efficient management of healthcare costs. | 262 |  | 3.61 | 0.84 |  | 1.15 | 6.87 | 35.11 | 43.51 | 13.36 |  | 56.87 |
|  | 1. KM-CP will not affect personalized care.^b^ | 262 |  | 2.55 | 0.93 |  | 10.69 | 42.75 | 29.39 | 15.65 | 1.53 |  | 17.18 |
|  | 1. KM-CP will not increase the risk of a KMD being sued unfairly.^b^ | 262 |  | 3.47 | 1.01 |  | 3.44 | 14.89 | 26.72 | 41.6 | 13.36 |  | 54.96 |
|  | 1. KM-CP is useful not only for nurses and other health workers, but also for KMDs.^b^ | 262 |  | 3.63 | 0.87 |  | 0.38 | 9.92 | 31.3 | 43.51 | 14.89 |  | 58.40 |
|  | 1. Autonomy and diversity in clinical processes will be maintained even with KM-CP.^b^ | 262 |  | 3.21 | 1.01 |  | 3.44 | 24.05 | 29.01 | 35.5 | 8.02 |  | 43.51 |
|  | 1. KM-CP is easy to use in hospitals and clinics.^b^ | 262 |  | 3.3 | 1.03 |  | 3.44 | 20.61 | 29.77 | 34.73 | 11.45 |  | 46.18 |
|  | 1. KM-CP will be used consistently in the future. ^b^ | 262 |  | 3.56 | 0.95 |  | 1.15 | 12.98 | 30.53 | 38.93 | 16.41 |  | 55.34 |
|  | 1. The administrative workload of KMDs will be similar even with KM-CP.^b^ | 262 |  | 2.75 | 1.07 |  | 10.3 | 34.73 | 30.92 | 17.56 | 6.49 |  | 24.05 |

Note:

1. Positive rate is defined as the sum of proportions of *positive* and *very positive*.
2. Questions 10–16 were analyzed after being rephrased to match the reverse-coded meaning of the original question. The original questions of 10–16 were as follows: 10. KM-CP increases the risk that patients with the same disease will receive the same treatment; 11. KM-CP increase the risk of a KMD being sued unfairly; 12. KM-CP is useful for nurses and other health workers, but not for KMDs; 13. KM-CP runs the risk of strictly standardizing care and making hospital management resemble a factory production line; 14. KM-CP can only be used in hospitals and is difficult to use in clinics; 15. KM-CP is a current trend that will soon disappear; 16. KM-CP will increase the amount of administrative documentation that KMDs need to perform.

KM-CP, Clinical pathways for Korean Medicine; KMD, Korean Medicine doctor; Std, Standard deviation.
